# The efficacy and safety of a nicotine conjugate vaccine (NicVAX^®^) or placebo co-administered with varenicline (Champix^®^) for smoking cessation: study protocol of a phase IIb, double blind, randomized, placebo controlled trial

**DOI:** 10.1186/1471-2458-12-1052

**Published:** 2012-12-06

**Authors:** Philippe HJ Hoogsteder, Daniel Kotz, Paul I van Spiegel, Wolfgang Viechtbauer, Ruth Brauer, Paul D Kessler, Matthew W Kalnik, Raafat EF Fahim, Onno CP van Schayck

**Affiliations:** 1 CAPHRI School for Public Health and Primary Care, Department of General Practice, Maastricht University, P.O. Box 616, Maastricht 6200, MD, The Netherlands; 2 Nabi Biopharmaceuticals, Rockville, Maryland, USA; 3 Department of Pulmonary Medicine, Slotervaart Hospital, Amsterdam, The Netherlands

**Keywords:** Nicotine vaccination, NicVAX, Varenicline, Combination therapy, Efficacy, Safety, Immunogenicity, Randomized controlled trial

## Abstract

**Background:**

A potential new treatment in smoking cessation and relapse prevention is nicotine vaccination which is based on active immunization against the nicotine molecule. This immunization will elicit the immune system to produce nicotine-specific antibodies that sequester nicotine in the blood stream, after inhaling tobacco products. The resulting antibody-antigen is too large to cross the blood–brain barrier and is therefore postulated to attenuate the rewarding effect of nicotine by preventing the latter from reaching its receptors in the brain and causing the release of dopamine. The aim of this paper is to describe the design of a phase IIb, multi-center, double blind, randomized, placebo controlled trial to assess the efficacy of the nicotine vaccine NicVAX^®^ co-administered with varenicline (Champix^®^) and intensive counseling as an aid in smoking cessation and relapse prevention.

**Methods/design:**

Two centers will include a total of 600 smokers who are motivated to quit smoking. At week −2 these smokers will be randomized, in a 1:1 ratio, to either 6 injections of NicVAX^®^ or placebo, both co-administered with 12-weeks of varenicline treatment, starting at week 0. The target quit day will be set after 7 days of varenicline treatment at week 1. Smokers will be followed up for 54 weeks. The primary outcome is defined as biochemically validated prolonged smoking abstinence from week 9 to 52. Secondary outcomes include safety, immunogenicity, smoking abstinence from week 37 to 52, abstinence from week 9 to 24, abstinence in the subset of subjects with the highest antibody response, and lapse/relapse rate.

**Discussion:**

This is the first study to assess the efficacy of a nicotine conjugate vaccine in combination with an evidence-based smoking cessation pharmacotherapy (varenicline) to quit smoking. Although NicVAX^®^ is primarily designed as an aid to smoking cessation, our study is designed to explore its potential to maintain abstinence and prevent relapse. The results of this trial will give a unique insight in the potential of nicotine vaccination for relapse prevention.

**Trial registration:**

ClinicalTrials.gov: (NCT00995033)

## Background

Tobacco use is the leading preventable cause of death in the world. Currently, about 1 billion people smoke of which eventually half will die from smoking-related diseases [1]. Worldwide, approximately 6 million people die from smoking each year, including more than 600,000 non-smokers who die from secondhand smoke [1]. Although smokers are generally aware of the health consequences of smoking they have major difficulties to stop smoking [2]. Without the use of effective therapies, less than 5% of the smokers are able to stay abstinent for one year after a quit attempt [3].

Nicotine is responsible for the addictive mechanism of tobacco use and the difficulties that smokers encounter when trying to quit smoking and remain abstinent. After inhalation of cigarette smoke, nicotine almost immediately travels to the blood stream and crosses the blood–brain barrier within 10 to 20 seconds [4]. At this point the nicotine molecule acts on several neurotransmitter systems where it activates α4β2 nicotinic acetylcholine receptors and the dopamine reward system responsible for the reinforcing and addictive effects of nicotine [5].

Pharmacotherapy treatment for smoking cessation focuses on minimizing the nicotine withdrawal effects by substitution of the nicotine reward effects or by attenuating the reinforcing effects of tobacco [6,7]. Currently available therapies such as nicotine replacement therapy (NRT), psychotropic drugs, and partial nicotine acetylcholine agonists, in combination with behavioral support, can increase abstinence rates to a maximum of 20 to 25% after one year [6,8-10]. NRT reduces withdrawal by replacement of nicotine, which might result from abrupt cessation of nicotine use [6] while psychotropic drugs, such as bupropion and nortriptyline cause a blockade of neuronal re-uptake of several neurotransmitters like norepinephrine, serotonine, and dopamine [8]. Blockage of this “biology of nicotine addiction” will reduce the reinforcing effect of nicotine and withdrawal symptoms [8]. A more recent developed treatment strategy is the use of partial nicotine acetylcholine agonists like varenicline and cytisine, which stimulate the release of sufficient dopamine to reduce craving and withdrawal while simultaneously acting as a partial antagonist by blocking the binding and consequent reinforcing effects of inhaled nicotine [11,12].

Since the majority of smokers who attempt to quit still fail to achieve long-term abstinence, and most smokers relapse in the first 8 days, the need for better cessation approaches is of major importance [3]. A potential new treatment approach in smoking cessation and relapse prevention is nicotine vaccination, in which smokers who want to quit smoking receive multiple injections of a nicotine conjugate vaccine.

### Nicotine vaccination: mechanism of action

Nicotine vaccination is a new therapy which is based on active immunization against the, otherwise, non-immunogenic, nicotine molecule. For this purpose, the small nicotine molecule is conjugated to a much larger carrier protein to induce and activate the immune system to produce highly specific nicotine antibodies [13]. These antibodies sequester nicotine in the blood stream, after inhaling tobacco products, and the resulting antigen-antibody molecule becomes too large to cross the blood–brain barrier [14]. By preventing large amounts of nicotine reaching the central nervous system, nicotine vaccination is believed to attenuate the rewarding effect of nicotine [15].

The nicotine conjugate vaccine NicVAX^®^, developed by Nabi Biopharmaceuticals, was initially developed as an aid in smoking cessation. For this purpose, multiple injections are administered prior to the planned quit date resulting in a gradual increase in anti-nicotine antibodies which helps smokers in gradually reducing the number of cigarettes and eventually achieve complete abstinence [16]. Nicotine vaccines could also be used to prevent relapse. Hence, vaccinated ex-smokers who lapse (i.e., take a puff of a cigarette, have a positive smoking status for 1 week after a period of abstinence) are expected to experience diminished reward from nicotine inhalation which could prevent a full blown relapse (i.e., a positive smoking status for at least 2 weeks after a period of abstinence) [17]. The effects of a brief exposure to a positive stimuli such as second hand smoke may also be blunted.

### Nicotine vaccination: safety

Three phase I/II clinical trials with NicVAX^®^ have been published so far. Two of these studies were designed to evaluate the immunogenicity and safety [18,19], while another study was especially designed to demonstrate the proof of concept to determine the relationship between immunogenicity and smoking cessation outcomes [16]. Regarding the reported safety data, participants commonly reported local reactions at the injection site like ‘ache’ and ‘tenderness’. The reported systemic reactions were mostly mild to moderate of intensity and included symptoms of general discomfort/malaise, myalgia, and headache [16]. There was no significant difference in local and systemic reactions between the placebo group and the NicVAX^®^ treatment group [16]. Of all reported safety data, there was only one serious adverse event (anaphylactic reaction) in a subject with a history of allergic reactions that was considered by the investigator to be treatment-related [16].

### Nicotine vaccination: immunogenicity

Immunogenicity has been shown to be dose-related. Additional injections and higher vaccine doses have been shown to be related to stronger immune responses and higher antibody titers [18]. These antibody titers typically peak following the final injection when 4 or 5 vaccinations are administered [16].The peak geometric mean antibody concentration reported in response to the nicotine conjugate vaccine NicVAX^®^ was 45 μg/ml in the subgroup who received 5 injections with 400 μg of NicVAX^®^[16]. Previous data reported no difference in antibody response between smokers and non-smokers [19].

### Nicotine vaccination: efficacy

Currently, only one study has been published to evaluate the relationship between smoking cessation outcomes and immunogenicity in smokers treated with 4 or 5 injections of NicVAX^®^ in different dose schedules [16]. A subgroup of the top 30% antibody responders were significantly more likely than the placebo group to achieve 8 weeks of continuous abstinence from weeks 19 through 26 (24.5% vs. 12.0%, odds ratio (OR) = 2.69, 95% confidence interval (CI) = 1.14–6.37) and weeks 19 through 52 (19.7% vs. 10.0%, OR = 2.64, 95%CI = 1.03–6.79). The target quit day was dependent on the dose regimen, which was either week 5 (for 5 injections schedule) or week 7 (for 4 injections schedule). Smokers who received the 5 injection schedule with 400 μg NicVAX^®^ dose elicited the highest antibody response, which resulted in significantly higher abstinence rates than placebo [16]. In vaccinated smokers who failed to quit smoking, the authors observed a statistically significant reduction in daily cigarette consumption between the top 30% antibody responders and the placebo group in weeks 19–52; the median reduction in cigarette consumption between both groups was 4.6 cigarettes a day [16]. There were no differences in withdrawal symptoms and no evidence for compensatory smoking in those smokers who received injections with NicVAX^®^[16].

During the conduct of the current study, Nabi Biopharmaceuticals announced negative results of two phase III, randomized, placebo controlled trials on the efficacy of NicVAX^®^[20]. Both trials were designed identically and included approximately 1,000 smokers who received a set of 6 injections of 400 μg NicVAX^®^ or placebo. The preliminary results of the trials showed that the primary endpoint of 16 weeks abstinence measured at 12 months was not met; there was no statistically difference between the NicVAX^®^ and placebo group [20].

### Nicotine vaccination: rationale for combination with varenicline

The purpose and rationale of the proposed study is to evaluate the efficacy of NicVAX^®^ co-administered with varenicline as an aid to smoking cessation, long-term abstinence, and relapse prevention. Preliminary data on NicVAX^®^ suggest that antibody levels peak after the last injection, and it is therefore likely that smokers who quit smoking have less difficulty to remain abstinent.

In the proposed study we combine NicVAX^®^ with a 12-week treatment of varenicline to stimulate participants to stop smoking before week 12. Varenicline is used for immediate smoking cessation by reducing withdrawal and craving during the first 12 weeks of treatment. Varenicline has proven to be superior to placebo for smoking cessation at one year of follow-up [9]. Therefore, this phase IIb study design will be ideal to evaluate relapse, especially because end of treatment abstinence with varenicline is about 45% from weeks 9 to 12 [11]. The current trial is designed so that the anti-nicotine antibody concentration at the end of the varenicline therapy (week 12) would be high enough to decrease the chance of relapse, occurring 2 weeks after the 4^th^ vaccination. It is worth noting that anti-nicotine antibodies in response to NicVAX^®^ do not cross react with varenicline.[Nabi unpublished data]

### Primary objective

The primary objective of our trial is to evaluate the efficacy of 6 injections with 400 μg of the nicotine conjugate vaccine NicVAX^®^ or placebo co-administered with varenicline for smoking cessation and relapse prevention, by comparing biochemically validated prolonged abstinence from week 9 to 52 in smokers who want to quit.

### Secondary objectives

There are several secondary objectives which aim to understand the benefit of NicVAX^®^ vaccination co-administered with varenicline for smoking cessation. The most important secondary objectives will be described in detail in this article (an overview of all other secondary objectives is listed in Appendix A).

1. To evaluate the safety and immunogenicity of NicVAX^®^.

2. To evaluate abstinence rates from week 37–52

3. To evaluate abstinence rates from week 9–24

4. Abstinence from week 9–52, week 9–24 and 37–52 in the subset of NicVAX subjects with the high antibody responses.

5. To evaluate lapse and relapse rates from week 12–52.

6. To evaluate withdrawal symptoms using the Minnesota Nicotine Withdrawal Scale (MNWS).

7. To evaluate nicotine dependency measured by the Fagerström Test for Nicotine Dependence (FTND).

8. To evaluate cigarette consumption.

### Ethical approval

The Dutch Central Committee on Research Involving Human Participants approved the study protocol (NL25046.000.08).

### Trial registration

This trial is registered at ClinicalTrials.gov (NCT00995033).

## Methods/design

### Study design

**Figure 1:**
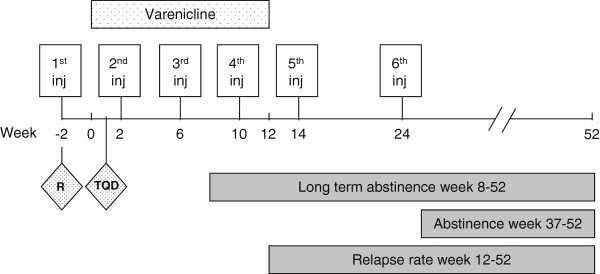
Study design overview.

In this phase IIb trial a total of 600 participants will be randomized in a 1:1 ratio to one of the 2 treatment groups, receiving either 400 μg NicVAX^®^ or matching placebo, both combined with open-label varenicline and intensive counseling (see Figure 1). After a 2-week screening period, in which the participant will be medically approved, the follow-up for each participant is 54 weeks. All participants will receive open-label varenicline starting at week 0 until week 12, together with a total of 6 injections in weeks −2, 2, 6, 10, 14, and 24. The target quit date will be set 1 week after starting the use of varenicline (3 weeks after randomization). Several quit re-challenges, where continuing smokers plan a new quit date, will be implemented at week 12, 16, and 26 for all participants who are not able to quit smoking successfully, corresponding to predicted elevations of anti-nicotine Ab levels occurring 2 weeks after the 4^th^, 5^th^, and 6^th^ vaccinations.

During follow-up, a total of 23 standardized behavioral counseling sessions based on motivational interviewing will be provided at the study sites (16 times) or via telephone (7 times). These counseling sessions aim to support participants in their quit attempt and are standardized to include the common elements of practical counseling (e.g., problem solving and skills training). Clinical and telephone counseling sessions will have a duration of approximately 10 minutes and will be performed by trained research nurses who are also responsible for the administration of the investigational product during the injection visits.

### Setting

This multicenter trial is coordinated by the CAPRHI School for Public Health and Primary Care and conducted at the Maastricht University Medical Centre and Slotervaart Hospital Amsterdam, the Netherlands.

### Study population

Smokers for the trial will be recruited through local media advertisements in the surrounding areas of Maastricht and Amsterdam and screened for eligibility on the telephone. Eligible participants will be sent written informed consent about the trial based on the principles of proper consenting. Major inclusion criteria will be: male or female between 18–65 years of age, being in good general health, smoking at least 10 cigarettes per day, and being motivated to quit smoking. The full inclusion and exclusion criteria for the trial are listed in Appendix B.

### Randomization and blinding

Eligible smokers will be randomly assigned to either the active nicotine vaccine group or the placebo group by the use of an Interactive Web Response System (IWRS). IWRS is based on a permuted block randomization list, where each randomization block is used by one center only. IWRS will assign an appropriate syringe number corresponding to the randomized treatment from the available syringes at the study center. Under no circumstance will a participant be injected with the investigational product from a syringe other than the one to which (s)he is assigned through IWRS. For proper blinding, we will use identical-appearing active and placebo vaccine syringes, which will be labeled with a unique numeric code, according to a randomized labeling list. The participants together with the involved investigators and sponsors will be blinded to the randomization lists until the clinical database is locked at the end of the trial.

### Sample size calculation

The primary end point of this study is biochemically validated prolonged abstinence from week 9 to week 52. The effect of a 12-week varenicline treatment on abstinence from smoking during 12 months has been shown to be about 22% [11,21]. A sample size of 300 smokers per group would provide 90% power to detect a difference in abstinence between the intervention and control groups of at least 12% (22% in the placebo group vs. 34% in the NicVAX^®^ group) with a 5% Type I error rate. As a consequence of the imputation analysis, where participants who become lost to follow-up will be included in the analysis as a smoker according to current practice in this field of research, the sample size is not corrected for lost to follow-up [22].

### Screening visit

After smokers have met the in- and exclusion criteria by telephone and agree on the written informed consent, the smokers will be invited for a screening visit. During this first clinical visit, we will obtain written informed consent and again review the in- and exclusion criteria. In addition, we will collect baseline demographic variables and vital signs, together with the currently used medications. In order to have background information on smokers’ smoking history, we will ask each subject to complete a tobacco use history report form with detailed information on several topics. A physician will obtain a medical history and a physical examination to medically approve each smoker before randomization. Chemistry, haematology, and urinalysis tests will be obtained for baseline values and to medically approve the general health status.

### Primary outcome measure

Our primary efficacy outcome for this study is defined as biochemically validated prolonged abstinence from week 9 to 52, measured by the participants’ self-reported smoking consumption on a weekly base and confirmed by exhaled carbon monoxide (CO) measurements in weeks 10, 12, 14, 18, 24, 28, 34, 40, 46, and 52, in the absence of use of NRTs during this entire period. If a CO measurement has been collected, it should confirm the smoking status of the participant for that week. A participant is considered to be a validated non-smoker with a CO level <10 ppm measured by a MicroCO (Micro Medical ltd., United Kingdom). When a participant is a self-reported non-smoker with an elevated CO level of ≥10 ppm, the CO-measurement supersedes the participants’ self-report and the participant is defined as a smoker.

### Secondary outcome measures

#### Safety

Local and systemic reactogenicity will be recorded on injection days (after 30 minutes and in the evening) as well as during the first 7 days after each injection. The participant has to report on the presence and severity of both local (erythema, swelling, burning, tenderness, ache, and heat) and systemic reactions (general discomfort/malaise, myalgia, headache, nausea, and vomiting) together with an oral temperature measurement. Local and systemic reactogenicity events starting 7 days post-injection will be recorded as adverse events. For the captured systemic reactogenicity, the study site physician will rate the relationship to the investigational product.

Adverse events (AE) and serious adverse events (SAE) will be collected from randomization through the final or early termination visit which are coded using the Medical Dictionary for Regulatory Activities (MedDRA) [23]. Assessment of AE and SAE’s relationship to the investigational product will be completed by a physician. The use of concomitant medications will be collected from 4 weeks prior to randomization though the entire study period.

During all clinical visits, we will measure vital signs (blood pressure, heart rate, respiratory frequency, and temperature) and for injection visits both prior to the injection and 25–35 minutes post injection.

Laboratory tests (haematology, chemistry, and urinalysis) will be performed at the screening visit and in week 6, 12, and 52 (Table 1).

**Table 1:** Scheduled clinical procedures

**Visits**	**1**	**2**	**3**	**4**	**5**	**6**	**7**	**8**	**9**	**10**	**11**	**12**	**13**	**14**	**15**	**16**	**17**	**18**	**19**	**20**	**21**	**22**	**23**	**24**	**25**
**Study Week**	-4 to -2	-2	-1	0	1	2	4	6	7	8	9	10	11	12	13	14	16	18	24	26	28	34	40	46	52/ET
**Study Day**	-28 to -14	-14	-7	0	7	14	28	42	49	56	63	70	77	84	91	98	112	126	168	182	196	238	280	322	364
**Visit Window** (**Days**)		±1	±1	±1	±2	±2	±2	±2	±2	±2	±2	±2	±2	±2	±2	±4	±4	±4	±4	±4	±5	±5	±5	±5	±5
Informed Consent	X																								
Inclusion/Exclusion Criteria	X																								
Medical History^1^	X	X																							
Tobacco Use History^1^	X	X																							
Randomize & Assign SID		X																							
Physical Exam^1^	X	X																							X
Vital signs and weight^2^	X	X		X	X	X	X	X		X		X		X		X		X	X		X	X	X	X	X
Exhaled Carbon Monoxide	X			X	X	X	X	X		X		X		X		X		X	X		X	X	X	X	X
Anti-Nicotine Antibody		X				X		X				X		X		X		X	X		X	X	X	X	X
Laboratory tests: Hematology, Chemistry	X							X						X											X
Urine Pregnancy Test	X^3^	X^3^				X		X				X				X			X						
Urinalysis	X							X						X											X
**QUIT DAY**					X																				
Quit Re-Challenge														X			X			X					
Clinic Counseling		X		X	X	X	X	X		X		X		X		X		X	X		X	X	X	X	
Telephone Counseling			X						X		X		X		X		X			X					
**NicVAX or Placebo**		X				X		X				X				X			X						
**Varenicline**				X	X	X	X	X	X	X	X	X	X	X											
Nicotine Exposure Report Form^4^	X	X	X	X	X	X	X	X	X	X	X	X	X	X	X	X	X	X	X	X	X	X	X	X	X
Minnesota Nicotine Withdrawal Questionnaire^5^	X	X												X	X	X				X	X				X
Reactogenicity Assessment^6^		X				X		X				X				X			X						
Fagerström Test for Nicotine Dependence (FTND)	X	X																	X						X
Adverse Events^7^		X	X	X	X	X	X	X	X	X	X	X	X	X	X	X	X	X	X	X	X	X	X	X	X
Concomitant Medications^8^	X	X	X	X	X	X	X	X	X	X	X	X	X	X	X	X	X	X	X	X	X	X	X	X	X

^1^ Medical History, Physical Exam and Tobacco Use History must be performed prior to randomization.

^2^Vital signs include blood pressure, heart rate, respiration, temperature, and weight. Vital signs will be taken immediately before injection and 30 ±5 minutes post injection.

^3^A negative urine pregnancy test is required for all females of childbearing potential within 3 days prior to IP injection. Females who have menstruated in the past 12 months (even if irregularly) or are not surgically sterile are considered to be of childbearing potential.

^4^ The Nicotine Use Report Form will be completed weekly for entire study. Blank Nicotine Use Report Forms should be provided to the subject at each visit.

^5^The Minnesota Nicotine Withdrawal Questionnaire is completed weekly during weeks 12–15, weeks 26–29 and weeks 49–52.

^6^Reactogenicity will be collected for 7 days after each injection. Blank MNWS forms should be provided prior to each collection period.

^7^Adverse Events will be collected thru day 364.

^8^Concomitant medications will be collected thru day 364.

#### Immunogenicity

Anti-nicotine IgG antibody concentrations in participants’ sera will be measured by Enzyme-linked immunosorbent assay (Elisa) at 13 time points as indicated in Table 1.

#### Abstinence from week 37–52

A secondary efficacy variable for this study is the biochemically confirmed 16-week abstinence at 12 months from weeks 37 to 52. This measurement is defined as the proportion of participants who maintained abstinence from weeks 37 to 52 measured by the weekly self-reports on smoking consumption, confirmed by exhaled CO measured at week 40, 46, and 52, and who also do not use NRTs during this period.

#### Abstinence from week 9–24

Defined as the proportion of subjects who maintained abstinence between weeks 9 to 24, inclusive, measured by subject self-report of smoking consumption on a weekly basis, confirmed by exhaled CO.

#### Abstinence from week 9–24, week 9–52 and 37–52 in high AB responders

Defined as biochemically confirmed 16-week abstinence at 6 months (week 9–24), 44-week abstinence at 12 months (week 9–52) and 16-week abstinence at 12 months (week 37–52) among the high antibody responders.

#### Lapse and relapse rate from week 12–52

Among participants with 4-week abstinence status at 12 weeks, we define relapse as at least 2 consecutive weekly self-reports that are positive for tobacco use. A lapse is defined as 1 weekly self-report for tobacco use followed and preceded by 1 weekly self-report for tobacco abstinence.

#### Withdrawal symptoms

Withdrawal symptoms will be measured 14 times by using the MNWS (item scores 0=“none” to 4=“severe”), reflecting craving for cigarettes, anger, irritability, frustration, anxiety, nervousness, depression, difficulty concentrating, insomnia, restlessness, and increased appetite or weight gain [24]. The MNWS will be collected at screening, weeks 12–15, weeks 26–29, and weeks 49–52.

#### Nicotine dependency

Nicotine dependency will be measured by the FTND and is administered at screening, week −2, 24, and week 52 as long as the participant continues to smoke [25].

#### Cigarette consumption

Information on cigarette consumption will be collected based on the participants’ self-reports on smoking status, in which they record their total cigarette consumption for the prior week.

### Data analysis

#### Data and Safety Monitoring Board (DSMB)

An independent DSMB will review all safety data available at pre-determined time points to decide on the continuation of the trial regarding the safety profile of the investigational product. We will use SPSS^®^ version 16.0 or higher to perform all statistical analyses.

#### Efficacy analysis

For our primary analysis we will compare NicVAX^®^ versus placebo, both co-administered with varenicline, in an intention-to-treat (ITT) analysis using a using a logistic regression model with corresponding 95% confidence interval, and p-value. The study has a single primary variable, and as such, no adjustments for multiple comparisons will be made. All statistical tests will be two-sided tests performed at a 0.050 level of significance.

#### Efficacy analysis in high AB responders

Subjects who receive NicVAX will be stratified based on peak antibody concentration (Cmax) and based on area under the curve. For each stratification scheme (AUC or Cmax) subjects will be placed into two or more percentile groups (50^th^, 33^rd^, 25^th^) based on AUC or Cmax. For each stratification method, the abstinence rate in the top antibody group compared with placebo will be initially performed first for the top 25^th^ percentile group. If the initial test is positive, the hypothesis will be retested for subsequent percentile groups (i.e., 33^rd^, 50^th^). The role of anti-nicotine antibodies will be established if the abstinent rate for the high responders (e.g. either 50^th^, 33^rd^, 25^th^ percentile) groups is superior to placebo.

#### Safety analysis

For each reactogenicity type, the presence of any post-vaccination reactogenicity, aggregated over all injections, will be summarized and compared between the treatment groups using Fisher’s exact test.

The numbers and percentages of participants experiencing AEs will be summarized by body system and preferred terms within a body system using the MedDRA dictionary. Summaries of AEs will also be generated by drug relationship and intensity. The maximum intensity and/or relationship for an AE within a patient will be used in these summaries. As appropriate, proportions of participants experiencing specific adverse events will be compared between the study treatments by Fisher’s exact test.

#### Immunogenicity analysis

Summary statistics including geometric mean concentration and corresponding 95% confidence interval, mean, minimum, maximum, 25^th^ and 75^th^ percentiles, standard deviation, and standard deviation in the log scale of antibody concentrations will be calculated for each time point. The immunogenicity data will be summarized on the ITT Analysis Set only.

#### Withdrawal symptoms analysis

We use baseline MNWS scores as the average of the scores over the screening and Day 0 visits. Follow-up MNWS scores for each participant will be averaged over non-missing weekly MNWS scores during each collection period (from 1–4 weeks during each collection period). Change from baseline on the average MNWS scores during each collection period will be analyzed with weighted analysis of covariance (weighted ANCOVA) models which will include the treatment group and center as factors and corresponding baseline MNWS score as a covariate. The number of non-missing weekly MNWS scores during each collection period (between 1 and 4 per participant) will be used as the weight. In addition, weekly MNWS scores collected throughout the study will be analyzed using mixed-effects repeated measures models. The overall treatment effect will be presented, along with the associated 95% confidence interval and p-value.

#### Nicotine dependency analysis

The baseline score is defined as the average of the scores collected during the screening and at week −2. A participant is considered as a smoker for completion of the FTND when smoking at the time of data collection, or if the participant has smoked cigarettes during the previous 7 days. Change from baseline in FTND at week 24 and 52 will be analyzed with similar ANCOVA (unweighted) and mixed-effects repeated measures models as described for the MNWS scores. The repeated measures analysis comparing NicVAX^®^ and placebo will be conducted for participants who are still smoking at the time of Fagerström data collection.

#### Cigarette consumption analysis

Baseline cigarette consumption is defined as the average of the screening visit and week −2.

A mixed-effects repeated measures analysis using the change from baseline data will be provided. The overall treatment effect will be presented, along with the associated 95% confidence interval and p-value. Change from baseline to week 24 and week 52, for those participants still smoking during week 26 and week 52 respectively, will be analyzed using ANCOVA, as described previously. The week 52 analysis will use the last available week’s data for completers who due to visit scheduling do not have week 52 data (e.g. week 51).

### Handling drop outs and missing data

Participants who withdraw from the study will be considered smokers for the remainder of the study. In the absence of CO measurement during a planned clinical visit, the confirmation on smoking status will be imputed using the CO measurements of both bordering visits. An abstinent status will only be assigned when both bordering visits are no more than one planned clinical visit apart and both CO measurements at these visits are < 10 ppm (i.e., participants are not allowed to miss two consecutive visits with CO measurements). In all other cases, a smoking status will be assigned. The refusal by a participant to undergo CO testing during a clinic visit will be considered to be an elevated CO level and the participant to be a smoker.

Any missing self-reports on smoking status will be imputed using both of the bordering non-missing weekly self reports. A missing self-report will be assigned an abstinent status only when both bordering self-reports have abstinent status. This imputation will only be carried out if the two bordering self-reports are no more than 4 weeks apart.

## Discussion

This will be the first study to evaluate the efficacy of a nicotine conjugate vaccine, NicVAX^®^, combined with varenicline as an aid in smoking cessation and relapse prevention. In addition, this is also the first trial to combine nicotine vaccination with an evidence-based smoking cessation pharmacotherapy. Although NicVAX^®^ is primarily designed as a smoking cessation aid, rather than a pharmacotherapy to prevent relapse in smokers who already quit, nicotine vaccination could be an ideal therapy to reduce the relapse rate.

As a consequence of the pharmacokinetics of active vaccination, it takes at least 4–5 injections with 400 μg NicVAX^®^ before a sufficiently high antibody concentration is acquired. Therefore, smokers who use NicVAX^®^ as smoking cessation therapy may gradually reduce their cigarette consumption before quitting. This process could take up to 16–26 weeks (5^th^ and 6^th^ injection) and could be a disadvantage for smokers who want to quit instantly. This concern may be reduced in the current protocol, which includes a small molecule with a rapid onset of action to allow for early quit attempts, prior to the end of varenicline treatment phase. Another potential shortcoming could be that smokers quit smoking before sufficient antibody titers are achieved and are therefore at increased risk to relapse into smoking. However, varenicline treatment is continued until the point that antibody levels are expected to be adequate. One of the advantages of nicotine vaccination is the increased treatment adherence compared to other cessation therapies due to the mode of administration, and the persistence of antibodies after vaccinations are completed, which may serve a role to maintain abstinence and prevent relapse. Because nicotine-specific antibodies will not interfere within the central nervous system, nicotine vaccination is also likely to have no adverse events related to interference with the central nervous system as in NRTs, antidepressants, and psychotropic medication.

Phase I/II trials assessing the immunogenicity of NicVAX^®^ concluded that continuous abstinence was closely related to the nicotine-specific immune response, in which the top 30% antibody responders had significantly higher abstinence rates than the placebo group. These results confirm the hypothesis that NicVAX^®^ and nicotine-specific antibodies could play a major role in smoking cessation.

Previously published studies on NicVAX^®^ as cessation therapy showed some disappointing results regarding its efficacy on the overall treatment groups. Although NicVAX^®^ was safe and well tolerated, preliminary results on the primary efficacy analysis of two large phase III studies, with a total of 2,000 participants who received 6 injections with 400 μg NicVAX^®^ or placebo, showed that continuous abstinence from week 37–52 was comparable between both treatment groups. At this point there are no published results of the secondary outcome measures of both phase III trials. Despite the disappointing phase III results of NicVAX^®^, we see a potential benefit for nicotine vaccination in future cessation programs, particularly in view of the observation in phase IIb studies that high antibody-responses correlates with increased cessation outcomes. This may be especially relevant if NicVAX^®^ is combined with other cessation aids such as varenicline and counseling.

This phase IIb trial is especially designed as a relapse prevention study, in which smokers who are motivated to quit smoking will be assisted with the use of a 12-week varenicline treatment. The study should allow us to gain more insight in NicVAX^®^ as combination therapy and its efficacy in relapse prevention.

## Appendix A. Secondary objectives

• To evaluate safety and immunogenicity,

• To evaluate long term abstinence from week 37–52,

• A bstinence from week 9–52 and week 37–52 in the subset of NicVAX subjects with the high antibody responses,

• To evaluate four week abstinence from week 9–12,

• To evaluate abstinence from week 37–52 for subjects who were intolerant to varenicline or who were non-abstinent from week 9–12,

• To evaluate point prevalence (7-day abstinence; PP) at 12, 26 and 52 weeks,

• To evaluate relapse rate post week 12 among subjects with 4-week Abstinence status at 12 week. Relapse is defined as at least 2 consecutive weekly diary entries for tobacco use.

• To evaluate abstinence from week 9–24.

• To evaluate time to first Relapse among subjects with 4-week Abstinence status at 12 week,

• To evaluate time to first Lapse among subjects with 4-week Abstinence status at 12 week. Lapse is defined as 1 weekly diary entry for tobacco use followed and preceded by 1 weekly diary entry for tobacco abstinence,

• To evaluate time between first Lapse and first Relapse among subjects with 4-week Abstinence status at 12 week,

• To evaluate withdrawal symptoms using Minnesota Nicotine Withdrawal Scale, measured at screening and during weeks 12–15, weeks 26–29, and weeks 49–52.

• Nicotine dependency measured by the Fagerström Test for Nicotine Dependence.

• To evaluate Cigarette consumption.

## Appendix B. Inclusion and exclusion criteria

### Inclusion criteria

a) Male or female, 18–65 years of age, who has provided written, informed consent, and who, in the opinion of the investigator, is likely to comply with all the requirements of the study.

b) Good general health.

c) Subjects must be smoking an average of at least 10 cigarettes per day during the past year and over the month prior to the screening visit, with no period of abstinence greater than 3 months in the past year.

d) If a female of child-bearing potential: a negative urine pregnancy test, and be willing to use acceptable birth control during study participation (oral, injectable, implantable contraceptive; intrauterine device; or barrier method with spermacide).

e) Alveolar carbon monoxide level ≥ 8 ppm.

### Exclusion criteria

a) Prior exposure to NicVAX^®^ or any other nicotine vaccine.

b) Any known allergic reaction to any components of the vaccine

c) Evidence or history of clinically significant allergic reactions. (Seasonal allergies allowed.)

d) Use of systemic steroids (>10 mg of prednisone or equivalent per day for >14 days), immunosuppressive agents or other medications within 30 days prior to administration of investigational product that might interfere with an immune response, or intention to use such medication within 30 days before or after subsequent investigational product doses. (The use of inhaled steroids is allowed.)

d) Known history of cancer or cancer treatment within 60 months prior to administration of investigational product, except for treated basal cell or squamous cell carcinoma.

e) Known infection with HIV, or congenital or other acquired immunodeficiency.

f) Known history of illicit drug or alcohol abuse or dependence (except nicotine) within 12 months prior to administration of investigational product and for the study duration.

g) Known history of serious psychiatric disorder within 3 months prior to administration of investigational product.

h) Required treatment for depression within the past 12 months.

i) History or current psychosis or bipolar disorder.

j) Current use of antidepressants, antipsychotics, mood stabilizers, naltrexone.

k) Clinically significant cardiovascular disease within the past 6 months. Examples of clinically significant cardiovascular disease would include the following: myocardial infarction, coronary artery bypass graft (CABG), percutaneous transluminal coronary angioplasty (PTCA), unstable angina, serious arrhythmia, or clinically significant ECG conduction abnormalities.

l) Hepatic or renal impairment.

m) Serious or unstable disease within the past 6 months, such as:

Uncontrolled hypertension.

Severe chronic obstructive pulmonary disease.

SGOT (AST) of SGPT (ALT) greater than 150% ULN or total bilirubin greater than 110% ULN.

Elevated serum creatinine.

Other clinically significant laboratory abnormality.

n) Body mass index >38 [calculated as weight (kg)/height^2^ (m)]

o) Known history of any condition or factor judged by the investigator to preclude participation in the study or which might hinder compliance.

p) Use of any smoking cessation treatment, such as over the counter or prescription nicotine replacement therapy (NRT), varenicline, bupropion, clonidine, nortryptyline, mecamylamine within 30 days of the first administration of investigational product, or intention to participate in any other nicotine-related modification strategy outside the scope of this protocol.

q) Intolerance to varenicline.

r) Use of tobacco products other than cigarettes, including pipe tobacco, cigars, snuff, and chew, or marijuana use within the past month and not agreeing to abstain from use of these products during study participation.

s) Use of Botox injections within 30 days prior to administration of investigational product and for the duration of the study.

t) Use of any investigational vaccine 30 days prior to each administration of study product (licensed vaccines may be administered at any time with the exception of one week prior to and one week after administration of investigational product).

u) Previous serious or unexpected adverse reaction to a vaccine, including Guillain-Barré syndrome.

v) Anticipated inability to fulfill all visits and examination procedures throughout the study period (approximately 54 weeks).

w) Receipt of an Investigational New Drug/Device 30 days prior to (or 5 half-lives, whichever is longer) administration of investigational product and for the duration of the study.

## Abbreviations

AB: Antibody; AE: Adverse events; weighted ANCOVA: weighted analysis of covariance; AUC: Area under the curve; Cmax: Peak antibody concentration; CI: Confidence interval; CO: Carbon monoxide; DSMB: Data and Safety Monitoring Board; Elisa: Enzyme-linked immunosorbent assay; FTND: Fagerström Test for Nicotine Dependence; ITT: Intention-to-treat; IWRS: Interactive Web Response System; MNWS: Minnesota Nicotine Withdrawal Scale; MedDRA: Medical Dictionary for Regulatory Activities; NRT: Nicotine replacement therapy; OR: Odds ratio; SAE: Serious adverse events.

## Competing interests

CAPHRI school for Public Health and Primary Care received a ZonMW TOP-grant for the conduction of this phase IIb trial. Paul D. Kessler, Matthew W. Kalnik and Raafat E.F. Fahim are employees of Nabi Biopharmaceuticals and have received salary support, stock, and stock options. Paul I. van Spiegel has received grants from and served as a speaker and consultant for Pfizer. All other authors were investigators on the clinical trial funded by Nabi Biopharmaceuticals and Pfizer.

## Authors’ contributions

All authors have made substantial contributions to conception and design, or acquisition of data; and have been involved in drafting the manuscript or revising it critically for important intellectual content; and have given final approval of the version to be published.

## Pre-publication history

The pre-publication history for this paper can be accessed here:

http://www.biomedcentral.com/1471-2458/12/1052/prepub
